# Predicting Deleterious Non-Synonymous Single Nucleotide Polymorphisms (nsSNPs) of *HRAS* Gene and In Silico Evaluation of Their Structural and Functional Consequences towards Diagnosis and Prognosis of Cancer

**DOI:** 10.3390/biology11111604

**Published:** 2022-11-02

**Authors:** Chuan-Yu Chai, Sathiya Maran, Hin-Yee Thew, Yong-Chiang Tan, Nik Mohd Afizan Nik Abd Rahman, Wan-Hee Cheng, Kok-Song Lai, Jiun-Yan Loh, Wai-Sum Yap

**Affiliations:** 1 Department of Biological Science, Faculty of Science, Universiti Tunku Abdul Rahman, Jalan Universiti, Bandar Barat, Kampar 31900, Malaysia; 2 School of Pharmacy, Monash University Malaysia, Jalan Lagoon Selatan, Bandar Sunway 47500, Malaysia; 3 School of Postgraduate Studies, International Medical University, Jalan Jalil Perkasa 19, Bukit Jalil, Kuala Lumpur 57000, Malaysia; 4 Department of Cell and Molecular Biology, Faculty of Biotechnology and Biomolecular Sciences, Universiti Putra Malaysia, 43400 Serdang, Selangor, Malaysia; 5 Faculty of Health and Life Sciences, INTI International University, Persiaran Perdana BBN, Putra Nilai, Nilai 71800, Malaysia; 6 Health Sciences Division, Abu Dhabi Women’s College, Higher Colleges of Technology, Abu Dhabi 41012, United Arab Emirates; 7 Centre of Research for Advanced Aquaculture (CORAA), UCSI University, No. 1, Jalan Menara Gading UCSI Height, Cheras, Kuala Lumpur 56000, Malaysia; 8 He & Ni Academy, Office Tower B, Northpoint Mid Valley City, Kuala Lumpur 59200, Malaysia

**Keywords:** HRAS, nsSNPs, RAS/MAPK signaling pathway, diagnosis, prognosis

## Abstract

**Simple Summary:**

The HRAS gene has been reported to cause cancer, and identifying alleles that could potentially predispose one to cancer could lead to early diagnosis and better prognosis. Here for the first time, we conducted a machine-learning approach to identify high-risk predictive alleles of the HRAS gene. Our study reported alleles that may serve as potential targets for different proteomic studies, diagnoses, and therapeutic interventions focusing on cancer.

**Abstract:**

The Harvey rat sarcoma (HRAS) proto-oncogene belongs to the RAS family and is one of the pathogenic genes that cause cancer. Deleterious nsSNPs might have adverse consequences at the protein level. This study aimed to investigate deleterious nsSNPs in the HRAS gene in predicting structural alterations associated with mutants that disrupt normal protein–protein interactions. Functional and structural analysis was employed in analyzing the HRAS nsSNPs. Putative post-translational modification sites and the changes in protein–protein interactions, which included a variety of signal cascades, were also investigated. Five different bioinformatics tools predicted 33 nsSNPs as “pathogenic” or “harmful”. Stability analysis predicted rs1554885139, rs770492627, rs1589792804, rs730880460, rs104894227, rs104894227, and rs121917759 as unstable. Protein–protein interaction analysis revealed that HRAS has a hub connecting three clusters consisting of 11 proteins, and changes in HRAS might cause signal cascades to dissociate. Furthermore, Kaplan–Meier bioinformatics analyses indicated that the HRAS gene deregulation affected the overall survival rate of patients with breast cancer, leading to prognostic significance. Thus, based on these analyses, our study suggests that the reported nsSNPs of HRAS may serve as potential targets for different proteomic studies, diagnoses, and therapeutic interventions focusing on cancer.

## 1. Introduction

The HRas proto-oncogene GTPase (HRAS) is one of the pathogenic genes that cause cancer. The human HRAS gene is located on chromosome 11p15.5 and is responsible for cell division by regulating the cellular signaling pathway using a molecular switch mechanism [1]. HRAS is encoded by the RAS protein, which is commonly deregulated in human cancer [2]. RAS is considered ‘undruggable’ because the RAS protein lacks a druggable binding pocket [3]. Mutations in HRAS have been reported in rhabdomyosarcoma, salivary gland carcinoma, thyroid carcinoma, and bladder and mouth carcinoma [4,5,6,7].

Single nucleotide polymorphisms (SNPs) account for more than 90% of all nucleic acid sequence variations in humans. Non-synonymous single nucleotide polymorphisms (nsSNPs) cause amino acid substitution, which may affect protein function and lead to pathogenic phenotypes [8]. nsSNPs have also been reported to affect protein stability, which impacts protein function and further alters a protein’s allosteric sites and its stability [9,10]. nsSNPs in genes are responsible for cell growth and could potentially lead to downregulation of the gene causing continuous proliferation, thus leading towards the formation of cancer cells and has been associated with various human diseases [11,12,13,14]. Furthermore, analyses of putative functional SNP in cancer genes have been reported to exhibit prominent improvement in human health in terms of personalized therapeutics [15].

**Figure 1:**
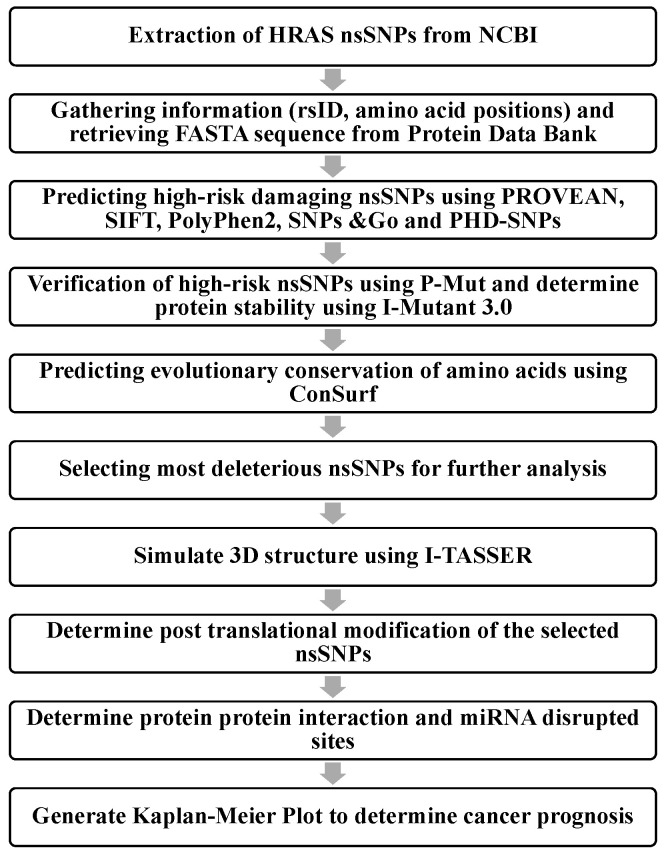
Flow chart of bioinformatics steps in this study.

Mutations in the HRAS gene have been reported to be common among different types of cancer; however, the structural and functional effect of the reported mutations remains vague. Understanding how the HRAS nsSNPs lead toward cancer could provide better insights into understanding the influencing pathways, which hold implications for cancer diagnosis and prognosis. Therefore, this study aims to investigate the nsSNPs of HRAS in understanding its pathogenesis for elucidating the diagnosis and prognosis of cancer using in silico analysis. The nsSNPs of the HRAS gene were extracted from the NCBI database and screened for high-risk pathogenicity using multiple bioinformatics software tools based on their high accuracy and frequency of use [16,17]. The various bioinformatics steps involved in this study are shown in Figure 1.

## 2. Materials and Methods

### 2.1. Retrieving nsSNPs

The HRAS nsSNPs were obtained from the NCBI dbSNP 2.0 (gene ID: 3265) database (National Center for Biological Information) (http://www.ncbi.nlm.nih.gov/ (accessed on 12 October 2020)). The amino acid and DNA sequences, SNP IDs, wild-type amino acids, amino acid positions, missense amino acids, and minor allele frequency (MAF) were also retrieved. A total of 180 nsSNPs were extracted from this database. The human HRAS protein structure was accessed from the Research Collaboratory for Structural Bioinformatics (RCSB) Protein Data Bank (PDB) (ID: 4Q21).

### 2.2. Identifying the Damaging nsSNPs

Five different bioinformatics tools were used to predict the functional effects of nsSNPs. These tools were: PROVEAN (Protein Variation Effect Analyzer) embedded with SIFT (Sorting Intolerant from Tolerant) [http://provean.jcvi.org/genome_submit_2.php?species=human (accessed on 18 October 2020)], PolyPhen-2 (Polymorphism Phenotyping v2) [http://genetics.bwh.harvard.edu/pph2/bgi.shtm (accessed on 18 October 2020), as well as SNPs&GO embedded with PhD-SNP (Predictor of human Deleterious Single Nucleotide Polymorphisms) [https://snps.biofold.org/snps-and-go/snps-and-go.html (accessed on 18 October 2020)]. The nsSNPs predicted deleterious by all five in silico tools were considered high-risk nsSNPs and investigated further.

### 2.3. Verifying the High-Risk nsSNPs

PMut [https://mmb.irbbarcelona.org/PMut/ (accessed on 20 August 2021)] was used to verify the nsSNPs identified by the previous five tools. The prediction has a range of 0–1, where the range of 0–0.5 is considered neutral while that of 0.5–1 is considered disease [18].

### 2.4. Analyzing Protein Stability

I-Mutant3.0 [http://gpcr2.biocomp.unibo.it/cgi/predictors/I-Mutant3.0/I-Mutant3.0.cgi (accessed on 28 October 2020)] was used to determine protein stability. Its predictions are based on two support vector machines (SVM), and it uses ProTherm, which is now the most comprehensive database of thermodynamic experimental data on protein stability when mutated (calculated as free energy change value DDG) [19]. It also predicts the reliability index (RI) of the results ranging from 0–10, where 10 is the highest reliability. Other than inputting the protein sequence and the mutation sites, the temperature and pH setting remained the same (25 °C, pH 7).

### 2.5. Analyzing Protein Evolutionary Conservation

ConSurf [https://consurf.tau.ac.il/ (accessed on 28 October 2020)] was used to estimate the evolutionary conservation of amino acid residues in a protein based on phylogenetic relations between homologous sequences [20]. In this study, 50 homologous sequences were used to estimate the conservation score of each residue of HRAS protein.

### 2.6. 3D Protein Modeling

The 3D models for wild-type HRAS protein and its mutants were generated using Phyre2 [http://www.sbg.bio.ic.ac.uk/~phyre2/html/page.cgi?id=index (accessed on 28 October 2020)] and I-TASSER (Iterative Threading ASSEmbly Refinement) [https://zhanglab.ccmb.med.umich.edu/I-TASSER/ (accessed on 5 November 2020)]. PDB formats for wild-type HRAS and its mutants were obtained through Phyre2 by substituting each nsSNP into the HRAS protein sequence [21]. Then, TM-align [https://zhanglab.ccmb.med.umich.edu/TM-align/ (accessed on 28 October 2020)] was employed to compare wild-type with mutant protein structures. This algorithm computes a template modeling score (TM-score) and the root mean square deviation (RMSD) along with the superposition of the structures. The TM-score gives values between 0 and 1, where 0.00 < TM score < 0.30 means random structural similarity, 0.50 < TM score < 1.00 means in about the same fold. While higher RMSD indicates greater variation between wild-type and mutant structures [22,23]. Finally, the proteins were subjected to I-TASSER, which predicts the top five highest possible protein models for the sequence. All the models generated were verified with ERRAT [https://servicesn.mbi.ucla.edu/ERRAT/ (accessed on 5 November 2020)], and the resulting structures were viewed using Chimera 1.15.

### 2.7. Predicting Post-Translational Modification (PTM) Sites

The putative methylation sites at lysine and arginine residues in the HRAS protein sequence were predicted using MusiteDeep [https://www.musite.net/ (accessed on 20 August 2021)] and GPS-MSP 1.0 [http://msp.biocuckoo.org/online.php (accessed on 20 August 2021)]. A local database from UnitProtKB/Swiss-Prot was always provided and updated in MusiteDeep. The known PTM sites were shown on the sequence according to the confidence threshold [8]. GPS-MSP 1.0 was used to predict the potential of methylation sites, and it only showed sites that were higher than the cutoff point [24]. Phosphorylation sites in the HRAS protein at serine, threonine, and tyrosine residues were predicted using NetPhos 3.1 [https://services.healthtech.dtu.dk/service.php?NetPhos-3.1 (accessed on 20 August 2021)] and GPS 5.0 [http://gps.biocuckoo.org/online.php (accessed on 20 August 2021)]. NetPhos 3.1 uses ensembles of neural networks to complete this task, and residues with scores >0.5 the residue was considered phosphorylated [25]. Likewise, in GPS 5.0, the positions with scores higher than the cutoff point were listed by the program [26]. Putative protein ubiquitylation sites at lysine residues were predicted by BDM-PUB [http://bdmpub.biocuckoo.org/prediction.php (accessed on 20 August 2021)] and iUbiq-Lys [http://www.jci-bioinfo.cn/iUbiq-Lys (accessed on 20 August 2021)]. In BDM-PUB, the Bayesian Discrimination Method (BDM) was used to determine the probability score [27]. In iUbiq-Lys, SVM was used as a vector for calculation [28].

### 2.8. Predicting Protein–Protein Interactions by Search Tool for the Retrieval of Interacting Proteins (STRING)

STRING (https://string-db.org/ (accessed on 3 December 2020)]) is a database that calculates protein–protein interactions [29]. The database contains data from empirical evidence, computational prediction tools, and collections of universal text. The interaction of HRAs proteins with other proteins was determined using STRING.

### 2.9. PolymiRTS Database 3.0

PolymiRTS (https://compbio.uthsc.edu/miRSNP/ (accessed on 3 December 2020)]) analyses SNPs and INDELs in microRNA, which may affect the miRNA-mRNA interactions resulting in altered expression of protein [30]. The effects of the variants are classified as “D” (the derived allele disrupts a conserved miRNA site), “N” (the derived allele disrupts a nonconserved miRNA site), “C” (the derived allele generates a new miRNA site), and “O” (the derived allele creates a new miRNA site). The ancestral allele cannot be determined with “D” and “C” groups indicating functional impacts.

### 2.10. Kaplan–Meier Plotter Analysis (KM Plotter)

The Kaplan–Meier plotter evaluates the impact of 54,000 genes on survival in 21 types of cancer using meta-analysis-based detection and validation of biomarkers for cancer [31]. The overall survival (OS) is the period of time from the start of changes in gene expression level until the time a patient diagnosed with it is still alive. A *p*-value less than 0.05 will be considered statistically significant.

### 2.11. Molecular Dynamics Simulation

The structural stability of the mutants was simulated in a temporal manner using molecular dynamics (MD) simulation for wild-type and each mutant structure using GROMACS version 2020 [32,33]. The MD simulation was powered by the CHARMM36 force field and CHARMM-modified TIP3P water model [34]. The systems were solvated with water contained in a dodecahedron box with borders a minimum of 1.0 Å from the protein structure. The system was deionized with default cations and anions to achieve electrostatic neutralization. To prepare the system for the MD environment, the system was minimized using the steepest descent algorithm until energy convergence. Then, NVT and NPT simulations for 100 picoseconds each to equilibrate the system at the proper starting temperature, pressure, and density were carried out. Finally, the system proceeded with the production of MD simulations for 10 nanoseconds (ns) to observe protein structure dynamics over time.

For analysis, the first and last frame of the protein throughout the production of the MD simulation, as well as the computed stability metrics such as root mean square deviation (RMSD), root mean square fluctuation (RMSF) per residue, and radius of gyration (Rg) were extracted.

## 3. Results

### 3.1. nsSNPs Retrieved from dbSNP Database

nsSNPs were retrieved from the dbSNP database as it is the more extensive SNP database [23]. A total of 2299 HRAS SNPs were extracted, of which 180 were nsSNPs located at the intronic region (28 nsSNPs) and exonic region (152 nsSNPs).

### 3.2. Deleterious nsSNPs Identified in HRAS Gene

PROVEAN, SIFT, PolyPhen-2, SNPs&GO, and PhD-SNP were used to predict and identify the most deleterious nsSNPs. A total of 33 nsSNPs were predicted as “pathogenic” or “harmful” by all five tools, hence they were high-risk nsSNPs (Table 1).

### 3.3. Verification of 33 HRAS High-Risk nsSNPs by PMut and I-Mutant

The 33 high-risk nsSNPs were further verified using PMut. Table 2 shows the prediction of the 33 nsSNPs by PMut for verification, where all of them were predicted to be “disease”, indicating that it is damaging. I-Mutant 3.0 was used to predict protein stability after each mutation. The effect on protein stability, reliability index (RI), and free energy change value (DDG) were predicted (Table 2). A decrease in stability was observed for 30 nsSNPs, of which V14G, I36T, F90S, L113P, and L133P showed a DDG value lower than −0.5, indicating a larger impact on the protein.

### 3.4. Conservation Profile of Deleterious nsSNPs in HRAS

ConSurf was used to calculate the evolutionary conservation of amino acid residues of the HRAS protein. V14, D38, T58, G60, R73, K117, E143, and A146 were predicted as functional, highly conserved, and exposed residues. G15 was predicted as structural, highly conserved, and buried residue. G12, G13, R102, R123, R161, and R164 were predicted as exposed but not functional residues. Y4, I36, M72, G75, G77, F90, L113, G115, A130, L133, A134, and I163 were predicted as buried residues (Table 3).

A total of eight: 38H, T58P, G60S, G60V, R73C, K117R, E143Q, and A146V nsSNPs were predicted to be highly conserved and exposed with decreased protein stability. These were confirmed as the most damaging and selected for comparative modeling.

### 3.5. Comparative Modeling of Wild-Type HRAS and Its Mutants

**Figure 2:**
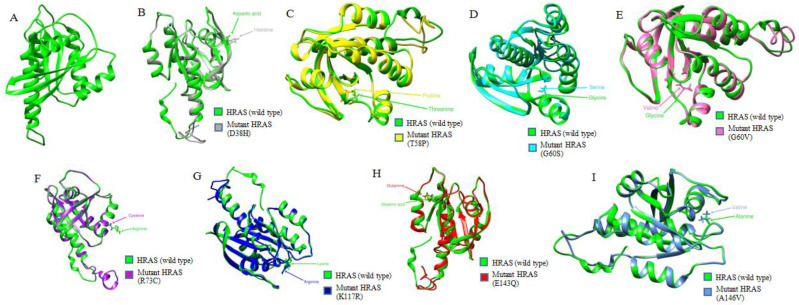
Comparison of wild-type HRAS protein structure with its mutant forms. (**A**) 3D model of wild-type HRAS protein. (**B**) Superimposed structures of wild-type HRAS protein and its mutant with mutation from Aspartic acid to Histidine at position 38. (**C**) Superimposed structures of wild-type HRAS protein and its mutant with mutation from Threonine to Proline at position 58. (**D**) Superimposed structures of wild-type HRAS protein and its mutant with mutation from Glycine to Serine at position 60. (**E**) Superimposed structures of wild-type HRAS protein and its mutant with mutation from Glycine to Valine at position 60. (**F**) Superimposed structures of wild-type HRAS protein and its mutant with mutation from Arginine to Cysteine at position 73. (**G**) Superimposed structures of wild-type HRAS protein and its mutant with mutation from Lysine to Arginine at position 117. (**H**) Superimposed structures of wild-type HRAS protein and its mutant with mutation from Glutamic acid to Glutamine at position 143. (**I**) Superimposed structures of wild-type HRAS protein and its mutant with mutation from Alanine to Valine at position 146.

To determine whether the eight high-risk nsSNPs alter the wild-type structure of HRAS protein, Phyre2 was used to generate 3D structures of the wild-type protein and its eight mutants. The c5c2kA template was used for predicting the 3D models. I-TASSER was used to generate the 3D models for the wild-type HRAS protein and mutants. The common templates used were 6cuoA, 5wdrA, 1c1y, 6zioA, 5tarA, and 1ctqA. For each input, five models were generated by I-TASSER, which was verified by ERRAT, and the model with the highest possible C-score and highest possible ERRAT value was selected (Table 4). The models were visualized with Chimera 1.15 (Figure 2).

### 3.6. Post-Translational Modifications

**Figure 3:**
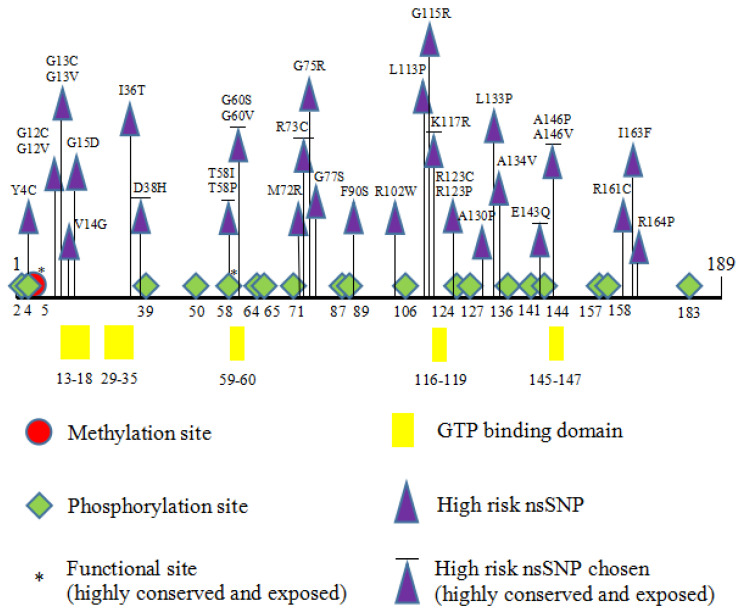
Putative PTM sites of high-risk nsSNPs in HRAS protein.

Post-translational modifications (PTMs) are biochemical modifications of amino acids that extend the structures and change the properties and functions of the protein, regulating the structural confirmation of proteins, protein–protein interactions, and cellular signaling processes [35]. Listing a few of many PTMs, methylation (N-methylation) occurs mainly on lysine and arginine residues; phosphorylation on serine, threonine, and tyrosine residues; and ubiquitylation on lysine residues only. The eight high-risk nsSNPs identified in this study were further investigated to see if they have any effect on PTMs in HRAS protein. The methylation predictors used were MusiteDeep and GPS-MSP 1.0. Only one residue (K5) was predicted by MusiteDeep (cutoff point = 0.5), while GPS-MSP predicted (at a very low cutoff point = 0) 11 lysine residues that can be methylated. The residue K5 was the only one in common between both tools (Figure 2). The phosphorylation sites in HRAS protein were predicted by NetPhos 3.1 and GPS 5.0. NetPhos 3.1 predicted a total of 19 residues (Ser:7, Thr:7, Tyr:5) that had the potential of being phosphorylated, while GPS 5.0 predicted 31 residues (Ser:11, Thr:11, Tyr:9). The common sites predicted by both tools are shown in Figure 3.

### 3.7. Protein–Protein Interaction Analysis

**Figure 4:**
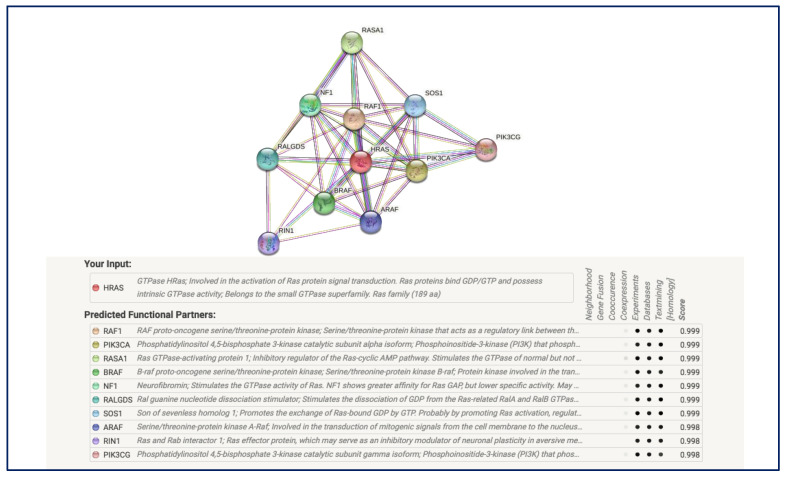
Protein–protein interaction network of HRAS with 10 partners.

Protein–protein interactions analysis was carried out to determine if the nsSNPs interact with other proteins, thus altering phenotypic effects. The interaction analysis showed that HRAS is related to RAS1, PIK3CA, RASA1, BRAF, NF1, RALGDS, SOS1, ARAF, RIN1, and PIK3CG gene (Figure 4). 

### 3.8. Prediction of nsSNPs within 3′ UTR

PolymiRTS database was used for the prediction of nsSNPs within 3′ UTR of the HRAS gene. nsSNP within the 3′ UTR region may disrupt and/or create miRNA target sites. A total of three: rs142218590, rs151229168, and rs140060409 functional SNPs were predicted to affect the miRNA target sites, further creating new miRNA binding sites (Table 4).

### 3.9. Expression Levels of HRAS on Overall Survival (OS) in Patients with Cancers

Kaplan–Meier plotter was used to determine the prognostic value of HRAS gene expression for breast, ovarian, lung, and gastric cancers by combining gene expression and cancer patient survival. Hazard ratio (HR) with 95% confidence intervals (CI) and log-rank *p*-value were calculated. HRAS gene showed a hazard ratio (HR) = 9.53 (95% CI: 1.3–69.88) and log-rank *p*-value = 0.006 for breast cancer; indicating that the result was statistically significant (the relation between the high expression of HLA-G gene and greater survival rate). However, no significant differences were observed for bladder carcinoma (*p* = 0.6591), cervical squamous cell carcinoma (*p* = 1), colon adenocarcinoma (*p* = 0.4552), cutaneous melanoma (*p* = 0.3256), head-neck squamous cell carcinoma (*p* = 0.5036), kidney renal clear cell carcinoma (*p* = 1), kidney renal papillary cell carcinoma (*p* = 1), lung adenocarcinoma (*p* = 9474), and ovarian cancer (*p* = 1). This indicates that HRAS deregulation can serve as a prognostic marker for patients’ breast cancer.

### 3.10. Visualization and Analysis of MD Simulation

RMSD, Rg, and RMSF per residue distributions using a Python library and Matplotlib were visualized and plotted [36]. The RMSF of each residue for each model via the ChimeraX defattr method was also visualized [37].

## 4. Discussion

The *HRAS* gene has been annotated in the cell proliferation of several carcinomas; however, the in silico analysis understanding of the structural and functional effect of deleterious nsSNPs has remained uncharacterized. The *HRAS* gene is reported to contribute to germline mutations that activate RAS/MAPK signaling to lead toward “RASopathies” [38]. HRAS is also reported as a frequently mutated gene in cancers, subsequently reported as an effective RAS inhibitor [39]. Therefore, any changes in the HRAS protein at functional and structural levels may alter its bio-molecular interactions. This study determined the impact of deleterious *HRAS* nsSNPs at molecular, functional, and structural levels using computational analysis to identify the most harmful nsSNPs and their impact on RASSF5 protein.

Damaging nsSNPs were predicted using five different tools (PROVEAN, SIFT, PolyPhen-2, SNPs&GO, and PhD-SNP), resulting in 33 nsSNPs predicted as “highly damaging”. In order to further narrow down the number of possible pathogenic nsSNPs, PMut, I-Mutant, and ConSurf tools were used to predict protein stability, the evolutionary conservation of amino acids, the physical and chemical properties, and the changes in protein structure after mutations. This resulted in eight “high-risk” nsSNPs: rs750680771, rs770492627, rs1589792804, rs730880460, rs749674880, rs104894227, rs909222512, and rs121917759, which were predicted (i) pathogenic by all five predicting tools; (ii) reduced protein stability; and (iii) evolutionary conservation showed these nsSNPs as highly conserved. This indicated that these nsSNPs with altered protein stability could cause misfolding, degradation, or aberrant conglomeration of proteins [40]. Moreover, we also found that these highly deleterious nsSNPs with high conservation scores could increase the risk of tumorigenesis by inactivating *HRAS*.

PTMs are essential in regulating the structures and functions of proteins and are involved in protein–protein interactions and cell signaling [41,42]. The eight “high-risk” nsSNPs were further investigated to determine their effect on PTM in the *HRAS* gene. The phosphorylation of *HRAS* at Y4C, T58I, and T58P was predicted to cause functional impairment leading to destabilization of the protein, eventually enhancing the harms of PTM impairment (Figure 3). As PTMs can modify the functions and regulate the expression of a protein, mutations in PTM regions can cause the regulation mechanism of the protein to malfunction, which will lead to the formation of cancer cells. Compared with ActiveDriverDB (https://www.activedriverdb.org/ (accessed on 3 December 2020)), the phosphorylation sites in Figure 4 matched the information. In a study by Ting and colleagues (2015), the authors found that phosphorylation of Y137 could enhance the signaling capacity of the HRAS protein by changing the protein conformation and effector binding [43]. While no methylation sites were shown on the website, it did show a few ubiquitination sites. However, only one (K170) predicted by BDM-PUB matched it. We followed the results using ubiquitination predictors and considered that no ubiquitination sites were predicted. The polarity and hydrophobicity of these three nsSNPs were also determined as it contributes to the protein’s structure and functionality. The prediction revealed that a polarity change was observed for T58I and T58P, indicating a change from neutral to hydrophobic and hydrophilic.

The structural consequences of these deleterious nsSNPs were predicted using the Phyre2 homology modeling tool. Six templates: 6cuoA, 5wdrA, 1c1y, 6zioA, 5tarA, and 1ctqA, were utilized to generate the wild-type and mutant protein models of the HRAS protein. Moreover, I-TASSER was used to calculate the C-score, which was verified by ERRAT. The model with the highest possible C-score and highest possible ERRAT value was selected. Based on these criteria, the eight nsSNPs were selected.

The protein interaction network of *HRAS* determined using the STRING tool showed a strong network (*RAF1*, *P1K3CA*, *RASA1*, *BRAF*, *NF1*, *RALGDS*, *SOS1)*, and a negative regulation of the fibroblast apoptotic process. *HRAS*, an upstream activated protein, binds to RAF and MEK kinases and transduces intra and extracellular signals tyrosine receptor kinases (Trk) in the MAPK/ERK pathway. Therefore, nsSNPs in *HRAS* may inhibit the MAPK/ERK pathway causing the restoration of tumor cells to a non-transformed state and increasing the activation of the ERK/MAPK signaling pathway leading to the occurrence and development of tumors [44].

microRNAs have been associated with cancer-associated biological processes, such as proliferation, differentiation, apoptosis, metabolism, invasion, metastasis, and drug resistance. Furthermore, the pathological origin of cancer has also been proven to be directly related to the dysregulation of miRNAs [45]. The polymiRTS analysis in this study showed that the rs142218590, rs151229168, and rs140060409 functional SNPs were predicted to affect the miRNA target sites, further creating new miRNA binding sites. Therefore, this might influence the regulation of HRAS, leading to pathological conditions [46].

This study also evaluated the *HRAS* nsSNPs against different types of cancer using the Kaplan–Meier bioinformatics analyses. The results indicated that the *HRAS* gene deregulation might affect the overall survival rate of patients with breast cancer and thus affect prognosis significance. This finding is in agreement with a study by Bieche and colleagues (2021) that reported that *HRAS*-mutated AMEs could potentially be treated with MEK inhibitors [47]. Another study by Zhumakayeva and colleagues (2019) reported the clinical applicability of *HRAS* as a prognostic factor or to serve as a therapeutic target for breast cancer treatment [48].

**Figure 5:**
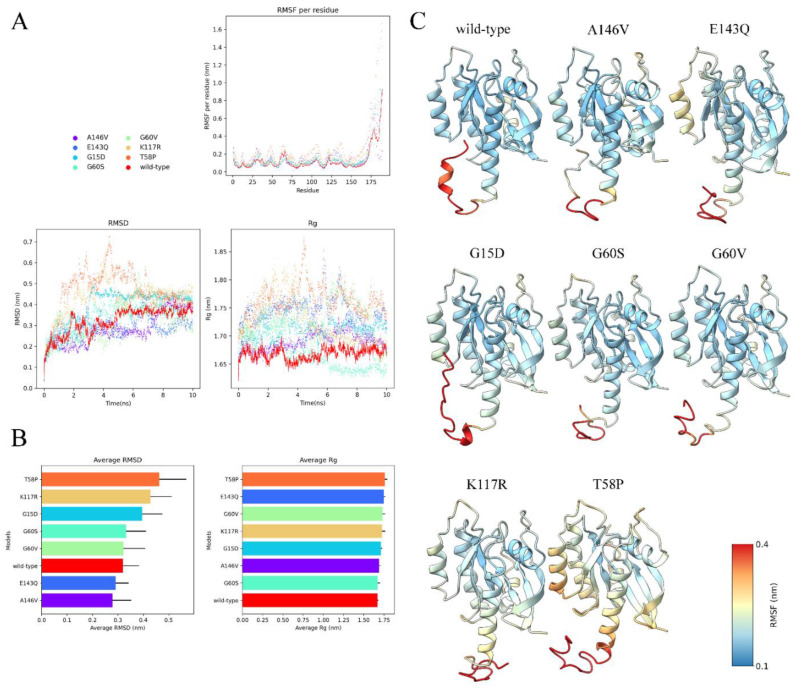
Results of MD simulation. (**A**) RMSD, Rg, and RMSF per residue of each model (wild-type, A146V, E143Q, G15D, G60S, G60V, K117R, and T58P). Herein, we observed overall structural stability in all models, except for their C-terminal coiled structure, based on low RMSD and RMSF values, as well as low Rg fluctuations. (**B**) The computed average RMSD and Rg values for each model over 10 ns to ease visualization. (**C**) Mapping of the RMSF values to their initial 3D structures to reveal the exact position of high- and low-fluctuating components.

Lastly, we performed 10 ns MD simulations for the wild-type HRAS and each mutant structure to simulate the structural stability of the mutant over time. All models were relatively stabilized without big structural fluctuations, as illustrated by the RMSD and RMSF scores of less than 1 nanometer (nm) (Figure 5). As expected, we observed relatively larger fluctuations at the C-terminus coiled structure unanimously for all models. Moreover, all models’ compactness remained stabilized per their invariant Rg values. Notably, T58P and K117R mutants consistently exhibited higher RMSD values, indicating more structural instability than others; however, most of their structural components, except for the C-terminal coils, fluctuated negligibly with RMSF of less than 0.4 nm.

## 5. Conclusions

The *HRAS* protein plays a vital role as a tumor suppressor. This study reports three major nsSNPs in *HRAS*: Tyrosine to Cysteine at position 4 (rs764622691), Threonine to Isoleucine at position 58 (rs121917758), and Threonine to Proline at position 58 (rs770492627) as high-risk. Furthermore, nsSNPs deregulation was also reported to affect the prognosis of breast cancer. These nsSNPs can be strongly considered as key molecular biomarkers for the diagnosis and prognosis of cancer. Nonetheless, in vitro studies are needed to explore the effects of these polymorphisms on the structure and function of the protein.

## Figures and Tables

**Table 1:** High-risk nsSNPs identified by five in silico programs.

SNP ID	Amino Acid Change	PROVEAN		SIFT		PolyPhen-2		SNPs&GO		PhD-SNP	
		Sc	(Cutoff = −2.5)	Pred	TI	Effect	Sc	Pred	RI	Pred	RI
rs764622691	Y4C	−6.39	Deleterious	Damaging	0	Pro-damaging	0.999	Disease	2	Disease	2
rs104894229	G12C	−7.26	Deleterious	Damaging	0.006	Pos-damaging	0.448	Disease	5	Disease	8
rs104894230	G12V	−7.21	Deleterious	Damaging	0.008	Pos-damaging	0.52	Disease	4	Disease	7
rs104894228	G13C	−7.72	Deleterious	Damaging	0	Pos-damaging	0.448	Disease	7	Disease	9
rs104894226	G13V	−7.65	Deleterious	Damaging	0	Pro-damaging	0.966	Disease	8	Disease	8
rs1589793707	V14G	−5.85	Deleterious	Damaging	0.001	Pro-damaging	1	Disease	2	Disease	7
rs1554885139	G15D	−5.66	Deleterious	Damaging	0.001	Pro-damaging	0.993	Disease	8	Disease	9
rs775056058	I36T	−3.57	Deleterious	Damaging	0.043	Pro-damaging	0.941	Disease	3	Disease	6
rs750680771	D38H	−6.02	Deleterious	Damaging	0	Pro-damaging	0.977	Disease	0	Disease	3
rs121917758	T58I	−5.82	Deleterious	Damaging	0	Pro-damaging	0.994	Disease	3	Disease	6
rs770492627	T58P	−5.82	Deleterious	Damaging	0.046	Pro-damaging	1	Disease	4	Disease	6
rs1589792804	G60S	−5.82	Deleterious	Damaging	0.001	Pro-damaging	0.959	Disease	4	Disease	6
rs730880460	G60V	−8.73	Deleterious	Damaging	0	Pro-damaging	0.997	Disease	5	Disease	7
rs755488418	M72R	−5.77	Deleterious	Damaging	0	Pos-damaging	0.873	Disease	2	Disease	7
rs749674880	R73C	−7.91	Deleterious	Damaging	0	Pro-damaging	0.97	Disease	5	Disease	9
rs756190012	G75R	−7.93	Deleterious	Damaging	0	Pro-damaging	0.999	Disease	5	Disease	8
rs1309567083	G77S	−5.94	Deleterious	Damaging	0.001	Pro-damaging	0.986	Disease	2	Disease	8
rs1589792507	F90S	−7.15	Deleterious	Damaging	0.006	Pro-damaging	0.997	Disease	3	Disease	8
rs1057517913	R102W	−6.82	Deleterious	Damaging	0	Pos-damaging	0.467	Disease	0	Disease	7
rs1389645747	L113P	−5.81	Deleterious	Damaging	0	Pro-damaging	0.986	Disease	5	Disease	8
rs917210997	G115R	−7.45	Deleterious	Damaging	0	Pro-damaging	0.99	Disease	5	Disease	7
rs104894227	K117R	−2.77	Deleterious	Damaging	0.004	Pro-damaging	0.964	Disease	0	Disease	5
rs369106578	R123C	−6.81	Deleterious	Damaging	0	Pro-damaging	0.99	Disease	3	Disease	8
rs730880464	R123P	−5.73	Deleterious	Damaging	0	Pro-damaging	1	Disease	6	Disease	8
rs1564788957	A130P	−3.27	Deleterious	Damaging	0.003	Pro-damaging	0.94	Disease	3	Disease	7
rs766801436	L133P	−4.91	Deleterious	Damaging	0.001	Pro-damaging	0.997	Disease	3	Disease	7
rs397517141	A134V	−3.49	Deleterious	Damaging	0.01	Pos-damaging	0.611	Disease	2	Disease	5
rs909222512	E143Q	−2.67	Deleterious	Damaging	0.009	Pos-damaging	0.765	Disease	1	Disease	4
rs104894231	A146P	−4.57	Deleterious	Damaging	0.001	Pro-damaging	0.994	Disease	6	Disease	7
rs121917759	A146V	−3.67	Deleterious	Damaging	0	Pos-damaging	0.596	Disease	4	Disease	7
rs758956556	R161C	−7.16	Deleterious	Damaging	0	Pro-damaging	1	Disease	3	Disease	7
rs1564787934	I163F	−3.57	Deleterious	Damaging	0.002	Pro-damaging	0.986	Disease	2	Disease	6
rs753977266	R164P	−4.66	Deleterious	Damaging	0.001	Pro-damaging	0.997	Disease	3	Disease	8

Pred = prediction; TI = tolerance index; Sc = score; Pro-damg = probably damaging; Pos-damg = possibly damaging; RI = reliability index.

**Table 2:** P-Mut, I-Mutant, and TM-align predictions for deleterious nsSNPs.

nsSNP ID	Amino Acid Change	PMut	I-Mutant		TM-Align Predictions		
		Prediction	Stability	RI	DDG (kcal/mol)	TM-Score	RMSD
rs764622691	4, Y→ C	0.75 (Disease)	Increase	0	−0.96	0.99193	0.54
rs104894229	12, G → C	0.79 (Disease)	Decrease	6	−1.20	0.99305	0.55
rs104894230	12, G → V	0.83 (Disease)	Decrease	4	−0.44	0.79612	2.03
rs104894228	13, G → C	0.82 (Disease)	Decrease	3	−1.17	0.99167	0.54
rs104894226	13, G → V	0.89 (Disease)	Decrease	5	−0.42	0.99167	0.54
rs1589793707	14, V → G	0.90 (Disease)	Decrease	10	−2.44	1	0
rs1554885139	15, G → D	0.90 (Disease)	Decrease	3	−0.80	0.99193	0.54
rs775056058	36, I → T	0.89 (Disease)	Decrease	9	−2.37	0.79612	2.03
rs750680771	38, D → H	0.90 (Disease)	Decrease	5	−0.48	0.98754	0.77
rs121917758	58, T → I	0.90 (Disease)	Increase	2	0.19	0.98201	0.93
rs770492627	58, T → P	0.90 (Disease)	Decrease	3	−0.33	0.99193	0.54
rs1589792804	60, G → S	0.90 (Disease)	Decrease	8	−1.13	0.99305	0.55
rs730880460	60, G → V	0.90 (Disease)	Decrease	6	−0.30	0.99167	0.54
rs755488418	72, M → R	0.90 (Disease)	Decrease	5	−0.87	0.80282	1.98
rs749674880	73, R → C	0.89 (Disease)	Decrease	3	−0.99	0.99193	0.54
rs756190012	75, G → R	0.85 (Disease)	Decrease	4	−0.39	0.98089	0.92
rs1309567083	77, G → S	0.90 (Disease)	Decrease	9	−1.49	0.99305	0.55
rs1589792507	90, F → S	0.90 (Disease)	Decrease	9	−1.99	0.98201	0.93
rs1057517913	102, R → W	0.85 (Disease)	Decrease	5	−0.41	0.97805	1.05
rs1389645747	113, L → P	0.89 (Disease)	Decrease	6	−1.71	0.79612	2.03
rs917210997	115, G → R	0.89 (Disease)	Decrease	3	−0.65	0.99193	0.54
rs104894227	117, K → R	0.90 (Disease)	Decrease	1	−0.20	0.97805	1.05
rs369106578	123, R → C	0.83 (Disease)	Decrease	5	−0.78	0.79612	2.03
rs730880464	123, R → P	0.90 (Disease)	Decrease	6	−0.58	0.99851	0.2
rs1564788957	130, A → P	0.90 (Disease)	Decrease	0	−0.16	0.98089	0.92
rs766801436	133, L → P	0.90 (Disease)	Decrease	5	−1.68	0.98649	0.72
rs397517141	134, A → V	0.89 (Disease)	Decrease	4	−0.14	0.99857	0.20
rs909222512	143, E → Q	0.89 (Disease)	Decrease	7	−0.61	0.98929	0.60
rs104894231	146, A → P	0.90 (Disease)	Increase	3	−0.04	0.98754	0.77
rs121917759	146, A → V	0.89 (Disease)	Decrease	2	0.07	0.98607	0.51
rs758956556	161, R → C	0.79 (Disease)	Decrease	6	−0.91	0.98459	0.56
rs1564787934	163, I → F	0.90 (Disease)	Decrease	7	−1.39	0.98754	0.77
rs753977266	164, R → P	0.80 (Disease)	Decrease	6	−0.75	0.98899	0.74

RI = reliability index; DDG = free energy change value; RMSD = root mean square deviation.

**Table 3:** ConSurf predictions showing conservation profile of amino acids in HRAS.

SNP ID	Residue and Position	Conservation Score	Prediction
rs764622691	Y4	5	Buried
rs104894229	G12	6	Exposed
rs104894230	G12	6	Exposed
rs104894228	G13	3	Exposed
rs104894226	G13	3	Exposed
rs1589793707	V14	9	Highly conserved and exposed (f)
rs1554885139	G15	9	Highly conserved and buried (s)
rs775056058	I36	8	Buried
rs750680771	D38	8	Highly conserved and exposed (f)
rs121917758	T58	9	Highly conserved and exposed (f)
rs770492627	T58	9	Highly conserved and exposed (f)
rs1589792804	G60	9	Highly conserved and exposed (f)
rs730880460	G60	9	Highly conserved and exposed (f)
rs755488418	M72	7	Buried
rs749674880	R73	8	Highly conserved and exposed (f)
rs756190012	G75	8	Buried
rs1309567083	G77	5	Buried
rs1589792507	F90	7	Buried
rs1057517913	R102	3	Exposed
rs1389645747	L113	7	Buried
rs917210997	G115	7	Buried
rs104894227	K117	9	Highly conserved and exposed (f)
rs369106578	R123	6	Exposed
rs730880464	R123	6	Exposed
rs1564788957	A130	2	Buried
rs766801436	L133	1	Buried
rs397517141	A134	8	Buried
rs909222512	E143	8	Highly conserved and exposed (f)
rs104894231	A146	9	Highly conserved and exposed (f)
rs121917759	A146	9	Highly conserved and exposed (f)
rs758956556	R161	3	Exposed
rs1564787934	I163	5	Buried
rs753977266	R164	3	Exposed

f = predicted functional residue (highly conserved and exposed); s = predicted structural residue (highly conserved and buried).

**Table 4:** Predicting the most deleterious missense nsSNPs of the protein isoforms of the HRAS gene.

dbSNP ID	Variant Type	miR ID	miRSite	Function Class	Context + Score Change
rs142218590	SNP	hsa-miR-6886-5p	agCTGCGGAagct	D	−0.22
		hsa-miR-1184	aGCTGCAGAagct	C	−0.386
		hsa-miR-1205	agCTGCAGAagct	C	−0.109
		hsa-miR-1301-3p	AGCTGCAgaagct	C	−0.187
		hsa-miR-17-3p	agCTGCAGAagct	C	−0.104
		hsa-miR-3158-5p	agCTGCAGAagct	C	−0.105
		hsa-miR-4660	AGCTGCAgaagct	C	−0.125
		hsa-miR-5047	AGCTGCAgaagct	C	−0.187
		hsa-miR-544a	agcTGCAGAAgct	C	−0.055
rs151229168	SNP	hsa-miR-6886-5p	aagCTGCGGAagc	D	−0.22
		hsa-miR-2115-5p	aagctgTGGAAGC	C	−0.137
		hsa-miR-3692-3p	aagcTGTGGAAgc	C	−0.077

## Data Availability

All data available in this manuscript.
